# An Assessment of the Measurement Equivalence of English and French Versions of the Center for Epidemiologic Studies Depression (CES-D) Scale in Systemic Sclerosis

**DOI:** 10.1371/journal.pone.0102897

**Published:** 2014-07-18

**Authors:** Vanessa C. Delisle, Linda Kwakkenbos, Marie Hudson, Murray Baron, Brett D. Thombs, the Canadian Scleroderma Research Group

**Affiliations:** 1 Department of Educational and Counselling Psychology, McGill University, Montreal, Quebec, Canada; 2 Department of Psychiatry, McGill University, Montreal, Quebec, Canada; 3 Department of Medicine, McGill University, Montreal, Quebec, Canada; 4 Department of Epidemiology, Biostatistics, and Occupational Health, McGill University, Montreal, Quebec, Canada; 5 Department of Psychology, McGill University, Montreal, Quebec, Canada; 6 School of Nursing, McGill University, Montreal, Quebec, Canada; 7 The Lady Davis Institute for Medical Research, Jewish General Hospital, Montreal, Quebec, Canada; University of Texas Health Science Center at Houston, United States of America

## Abstract

**Objectives:**

Center for Epidemiologic Studies Depression (CES-D) Scale scores in English- and French-speaking Canadian systemic sclerosis (SSc) patients are commonly pooled in analyses, but no studies have evaluated the metric equivalence of the English and French CES-D. The study objective was to examine the metric equivalence of the CES-D in English- and French-speaking SSc patients.

**Methods:**

The CES-D was completed by 1007 English-speaking and 248 French-speaking patients from the Canadian Scleroderma Research Group Registry. Confirmatory factor analysis (CFA) was used to assess the factor structure in both samples. The Multiple-Indicator Multiple-Cause (MIMIC) model was utilized to assess differential item functioning (DIF).

**Results:**

A two-factor model (Positive and Negative affect) showed excellent fit in both samples. Statistically significant, but small-magnitude, DIF was found for 3 of 20 CES-D items, including items 3 (*Blues*), 10 (*Fearful*), and 11 (*Sleep*). Prior to accounting for DIF, French-speaking patients had 0.08 of a standard deviation (SD) lower latent scores for the Positive factor (95% confidence interval [CI]−0.25 to 0.08) and 0.09 SD higher scores (95% CI−0.07 to 0.24) for the Negative factor than English-speaking patients. After DIF correction, there was no change on the Positive factor and a non-significant increase of 0.04 SD on the Negative factor for French-speaking patients (difference = 0.13 SD, 95% CI−0.03 to 0.28).

**Conclusions:**

The English and French versions of the CES-D, despite minor DIF on several items, are substantively equivalent and can be used in studies that combine data from English- and French-speaking Canadian SSc patients.

## Introduction

Systemic sclerosis (SSc, or scleroderma) is a chronic, multi-system connective tissue disorder characterized by vasculopathy, thickening and fibrosis of the skin, involvement of internal organs, significant morbidity and mortality, and substantially reduced quality of life [1]–[3]. A recent study of 345 SSc patients reported that 30-day, 12-month, and lifetime rates of major depressive disorder were 4%, 11%, and 23%, respectively [4]. A systematic review found that 36–65% of patients with SSc report high levels of emotional distress based on scores on depression symptom questionnaires [5], and qualitative interviews have confirmed that emotional distress is an important concern for people with SSc, whether or not they meet criteria for a psychiatric diagnosis [6].

The Center for Epidemiologic Studies Depression (CES-D) Scale, a 20-item questionnaire that was originally developed in the United States to measure depressive symptoms in the general population [7], is by far the most commonly used measure of symptoms of depression in SSc [5]. Published studies have used the CES-D with SSc patients in English [8]–[13], French [10]–[13], Dutch [14], and German [15]. The availability of the CES-D in multiple languages is important because international multi-center collaborations, in which outcomes are reported by patients in multiple languages, are frequently utilized in rare diseases such as SSc. In addition, in countries with more than one common language, such as Canada (French and English) or the United States (Spanish and English), outcomes reported in more than one language are commonly obtained and combined in analyses. However, results from measures administered in different linguistic or cultural settings can only validly be pooled if it has been established that the measurement metric is equivalent across versions of the measure [16], meaning that scores are not influenced by linguistic or cultural differences, in addition to the construct being measured. When the measurement metric is equivalent, patients across language groups with similar levels of an outcome construct (e.g., depression) should have similar scores on items measuring the construct (e.g., CES-D items). Differential item functioning (DIF) is said to occur when translation has altered the item’s meaning or when cultural factors influence interpretation of an item, leading to responses that differ across groups even when levels of the outcome construct being measured are similar [17]. Since scores on the CES-D are summed to obtain a total score, a finding that there is not meaningful DIF would establish that scores across language groups are equivalent metrically.

One study [18] assessed the metric equivalence of the CES-D in English- and Dutch-speaking patients with SSc and found that there was statistically significant DIF for 3 items, but that DIF was minor and that overall depression scores were not influenced substantively by DIF on these items. No other studies, however, have assessed the degree to which different language versions of the CES-D are metrically equivalent in medical populations. In Canada, studies of patients with SSc routinely administer the CES-D in English and French and pool scores across language versions [10]–[13]. However, no study has examined whether English and French versions of the CES-D are metrically equivalent.

The objective of this study was to assess the equivalence of scores on English and French versions of the CES-D in patients with SSc.

## Methods

### Ethics Statement

The sample of this study consisted of patients with SSc enrolled in the Canadian Scleroderma Research Group Registry (CSRG). This study was approved by the Institutional Review Board of McGill University. All patients provided informed written consent.

### Patients and Procedures

The study included patients who completed the CES-D from September 2004 through February 2012. Patients in the CSRG Registry are recruited from 15 centers across Canada and are eligible for enrolment if they are at least 18 years of age, fluent in English or French, and have been diagnosed with SSc by a Registry rheumatologist. Over 98% of patients in the Registry meet the 2013 ACR/EULAR classification criteria for SSc [19], [20]. At enrolment and annually thereafter, patients undergo extensive physical examinations and complete a series of self-report questionnaires in their preferred language (English or French). For patients who completed the CES-D at multiple annual assessments, only data from the first available visit with complete CES-D item responses were included in the present study.

### Measures

#### Sociodemographic and disease-related variables

Self-reported sociodemographic variables included sex, age, education level (post-secondary versus no post-secondary education), employment status (currently employed versus unemployed), and marital status (married or living as married versus unmarried). Disease-related variables were assessed by study physicians and included disease duration, disease subtype, and modified Rodnan skin score. Disease duration was defined as the time since the onset of the first non-Raynaud’s disease manifestation. Limited SSc was defined as skin involvement distal to the elbows and knees with or without face involvement, and diffuse SSc was defined as skin involvement proximal to the elbows and knees and/or involving the trunk [21]. The extent of skin involvement was assessed using the modified Rodnan skin score, which is a standardized rating of skin involvement ranging from 0 (*“No involvement”*) to 3 (*“Severe thickening”*) in 17 body areas [22].

#### Symptoms of depression

The CES-D [7] is a 20-item self-report measure that assesses the frequency of depression symptoms over the past week on a 0–3 Likert-type scale (*“Rarely or none of the time” to “Most or all of the time”)*. Items 4, 8, 12, and 16 are reversed scores, and total scores range from 0 to 60. Standard cutoffs are ≥16 for “possible depression” and ≥23 for “probable depression” [7]. The original English version of the CES-D [7] has shown to be a reliable and valid measure of depressive symptoms in patients with SSc [11]. The French version of the CES-D [23], which was designed for use in France, was adapted for use in Quebec, Canada, by a professional translator (see Figure S1).

### Statistical Analyses

Sociodemographic and disease-related variables were compared between English- and French-speaking patients using chi-square tests for categorical variables and t-tests for continuous variables.

Ideally for DIF assessment, the simplest structure with reasonable fit is used. In previous studies in patients with SSc, a two-factor model representing ‘positive affect’ (items 4, 8, 12, and 16) and ‘negative affect’ (all other items) has been reported [11], and this model was used to test for DIF in a previous study of English- and Dutch-speaking patients [18]. Thus, we assessed whether this two-factor model fit the data reasonably well in both samples separately using confirmatory factor analysis (CFA) with Mplus. Item responses for the CES-D Scale are ordinal Likert data, so the weighted least squares estimator with a diagonal weight matrix, robust standard errors, and a mean- and variance-adjusted chi-square statistic was used with delta parameterization [24]. Modification indices were used to identify pairs of items within scales for which model fit would improve if error estimates were freed to covary and for which there appeared to be theoretically justifiable shared method effects (e.g., similar wording) [25]. To assess model fit, the chi-square test, the Tucker-Lewis Index (TLI) [26], the Comparative Fit Index (CFI) [27], and the Root Mean Square Error of Approximation (RMSEA) [28] were used. Since the chi-square test is highly sensitive to sample size, it can lead to the rejection of well-fitting models [29]. Therefore, the TLI, CFI and RMSEA fit indices were emphasized. Good fitting models are indicated by a TLI and CFI≥0.95 and RMSEA≤0.06 [30]. Once the factor structure was established for English- and French-speaking patients separately, a CFA model was fit that included both English- and French-speaking patients.

To determine if the CES-D Scale exhibited DIF for English- versus French-speaking patients, the Multiple-Indicator Multiple-Cause (MIMIC) model was utilized. MIMIC models for DIF assessment are based on structural equation models, in which the group variable (English/French) is added to the basic CFA model as an observed variable. Thus, the base MIMIC model consists of the CFA factor model with the additional direct effect of group on the latent factors, which serves to control for group differences on the level of the latent factors. An important strength of the MIMIC model is that it allows for adjustment for covariates that may differ between comparison groups by adding a direct effect of these variables on the latent factors. Thus, we controlled for differences between samples on sociodemographic and disease-related variables, by adding a direct effect on the latent factors for sex, age, education level, employment status, marital status, disease duration, disease subtype, and skin score.

**Figure 1:**
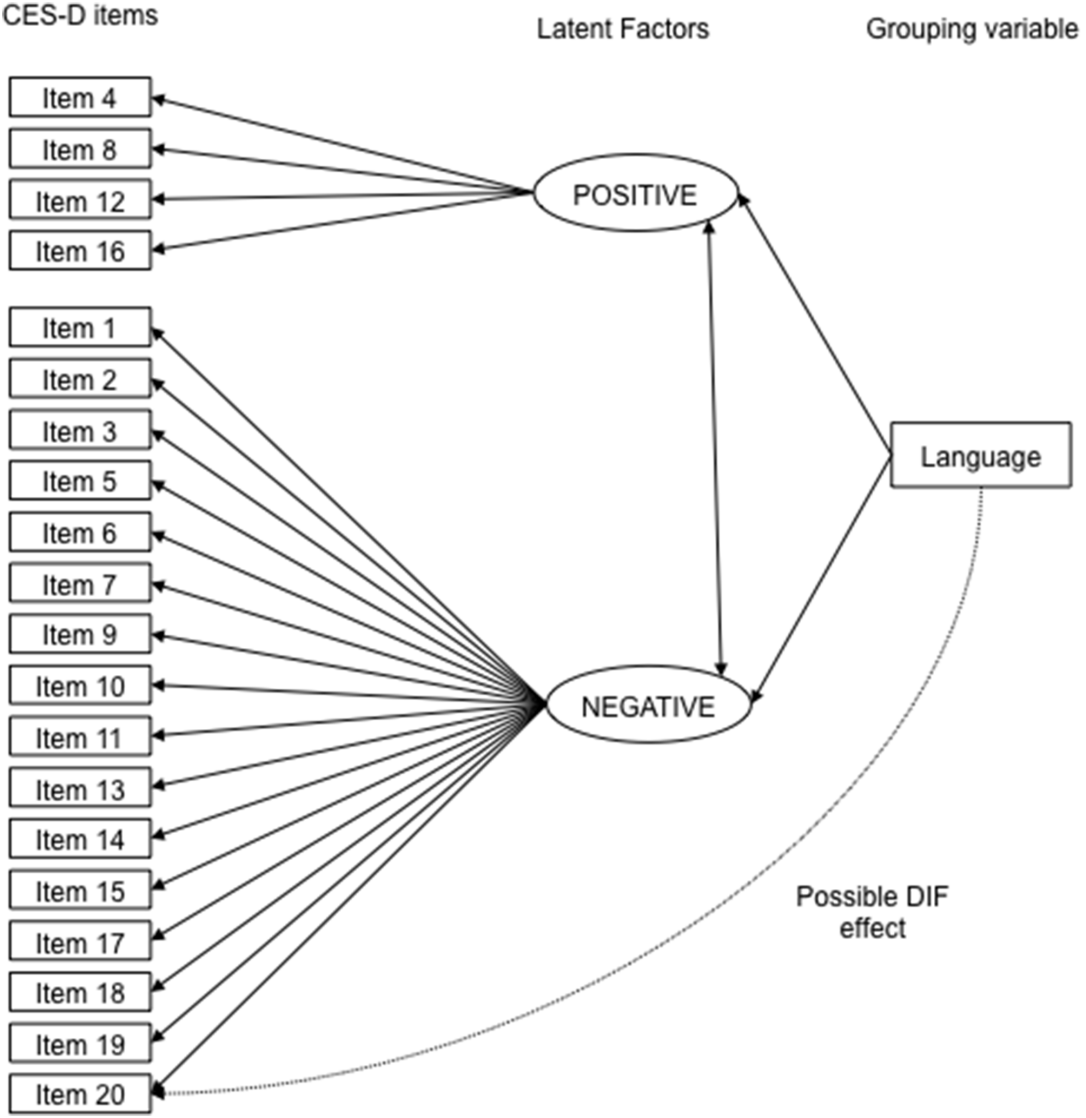
MIMIC Model of the CES-D.

To assess potential DIF, the direct effect of group on CES-D Scale items was assessed for each item separately, by regressing the items, one at a time, on group (see Figure 1). Each item was tested separately to determine if there was statistically significant DIF. Statistically significant DIF is represented by a statistically significant association in the model from language to the item, while controlling for any differences in the overall level of the latent factor between groups (by regressing the latent factor on language). If there was DIF for one or more items, the item with the largest magnitude of DIF was considered to have DIF, and the link between the group variable and that item was included in the model. Then, this procedure was repeated until none of the remaining items showed significant DIF. Hommels’ correction for multiple testing [31] was applied. Once all items with significant DIF were identified, the potential magnitude of DIF items collectively was evaluated by comparing the difference on the latent factor between groups in the baseline CFA model and after controlling for DIF. The magnitude of this difference was interpreted following Cohen’s effect sizes, with ≤0.20 standard deviation (SD) indicating small, 0.50 SD = moderate and 0.80 SD = large differences [32]. All CFA and DIF analyses were conducted using Mplus [24] and all other analyses were conducted using IBM SPSS Statistics 20 (Chicago, IL).

## Results

### Sample Characteristics

Demographic and disease characteristics for both samples are displayed in Table 1.

**Table 1:** Sociodemographic and Disease-Related Characteristics.

Variable	English-speaking patients (N = 1007)	French-speaking patients (N = 248)	P-value
**Sociodemographic characteristics**			
Female, n (%)	856 (85.0)	220 (88.7)	0.14
Age in years, mean (SD)	55.4 (12.4)^a^	57.5 (10.4)	0.01
Post-secondary education, n (%)	496 (49.5)^b^	99 (39.9)	0.01
Employed, n (%)	425 (42.3)^c^	99 (39.9)	0.49
Married or living as married, n (%)	837 (83.1)	201 (81.0)	0.44
**Disease-related characteristics**			
Limited disease, n (%)	618 (61.4)	153 (61.7)	0.93
Disease duration, mean (SD)	10.9 (9.3)^d^	9.5 (9.7)^e^	0.04
Modified Rodnan skin score, mean (SD)	10.2 (9.5)^f^	10.5 (9.7)^g^	0.67
**CES-D Scale characteristics**			
Total score, mean (SD)	14.4 (10.4)	15.0 (10.7)	0.41

Due to missing values:

a ^a^n = 1005;

b ^b^n = 1003;

c ^c^n = 1004;

d ^d^n = 992;

e ^e^n = 247;

f ^f^n = 991; and ^g^n = 243.

#### English-speaking sample

The English sample consisted of 1007 patients who completed the CES-D, with a mean age of 55.4 years (SD = 12.4) and mean disease duration of 10.9 years (SD = 9.3). The majority (85.0%) were female and most patients were married or living as married (83.1%). Most patients (61.4%) had limited SSc. The mean CES-D score was 14.4 (SD = 10.4).

#### French-speaking sample

In total, 248 patients completed the CES-D in French. The mean age was 57.5 years (SD = 10.4) and the mean disease duration was 9.5 years (SD = 9.7). The majority of patients (88.7%) were female and were married or living as married (81.0%). Most patients (61.7%) had limited SSc. The mean CES-D score was 15.0 (SD = 10.7).

English-speaking patients were significantly younger than French-speaking patients, were significantly more likely to have completed some post-secondary education, and had significantly longer disease duration than French-speaking patients (P<0.05).

### Confirmatory Factor Analysis

A two-factor structure was assessed for English- and French-speaking samples separately. The model showed good fit to the data in both samples (English-speaking sample: χ^2^(169) = 957.9, P<0.001, CFI = 0.96, TLI = 0.96, RMSEA = 0.07; French-speaking sample: χ^2^(169) = 326.7, P<0.001, CFI = 0.97, TLI = 0.97, RMSEA = 0.06). In both samples, inspection of modification indices indicated that freeing error terms to covary for items 7 and 20, 15 and 19, and 17 and 18 would improve model fit, and in each case there was clearly recognizable overlap in item content. Therefore, the model was refitted to the data, allowing the error terms of those items to be correlated. This change resulted in a model with excellent fit to the data in both samples (English-speaking sample: χ^2^(166) = 634.2, P<0.001, CFI = 0.98, TLI = 0.97, RMSEA = 0.05; French-speaking sample: χ^2^(166) = 245.6, P<0.001, CFI = 0.98, TLI = 0.98, RMSEA = 0.04).

### Differential Item Functioning

The two-factor structure was fit to the combined English- and French-speaking sample, including a direct effect of language (English/French) and covariates on the latent factors to correct for differences in latent depression levels between the samples and differences in sample characteristics, respectively. The two-factor model continued to have an excellent fit (χ^2^(102) = 1032.5, P<.001, CFI = 0.97, TLI = 0.97, RMSEA = 0.04). Table 2 shows the baseline CFA model parameters, with data from both samples, prior to assessing DIF. Prior to accounting for DIF, French-speaking patients had 0.08 SD lower latent scores for the “positive” factor (95% confidence interval [CI]−0.25 to 0.08) and 0.09 SD higher scores for the “negative” factor than English-speaking patients (95% CI−0.07 to 0.24), although neither difference was statistically significant. Three items showed significant DIF: items (*Blues*), 10 (*Fearful*), and 11 (*Sleep*). Specifically, French-speaking patients had higher scores on item 10 (z = 4.0, P<0.001) than English-speaking patients, while French-speaking patients had lower scores on items 3 (z = –5.6, P<0.001), and 11 (z = –3.9, P<0.001) than English-speaking patients (Table 2).

**Table 2:** Factor loadings for the “Positive” and “Negative” latent factors of the CES-D and influence on the overall estimates of fatigue latent factor scores.

	Base model^a^		DIF correctedmodel^b^	
	Factor loading	95% CI	Factor loading	95% CI
**Positive factor items:**				
4. Good	0.59	[0.54, 0.64]	0.59	[0.54, 0.64]
8. Hopeful	0.75	[0.71, 0.78]	0.75	[0.71, 0.79]
12. Happy	0.89	[0.85, 0.93]	0.89	[0.85, 0.93]
16. Enjoy	0.88	[0.84, 0.91]	0.88	[0.84, 0.91]
**Negative factor items:**				
1. Bothered	0.72	[0.68, 0.76]	0.72	[0.68, 0.76]
2. Appetite	0.51	[0.46, 0.57]	0.51	[0.46, 0.57]
3. Blues	0.85	[0.82, 0.88]	0.85	[0.82, 0.88]
5. Mind	0.70	[0.66, 0.74]	0.70	[0.66, 0.74]
6. Depressed	0.90	[0.88, 0.92]	0.90	[0.88, 0.92]
7. Effort	0.68	[0.64, 0.71]	0.68	[0.64, 0.71]
9. Failure	0.78	[0.74, 0.82]	0.78	[0.74, 0.82]
10. Fearful	0.69	[0.65, 0.74]	0.69	[0.65, 0.73]
11. Sleep	0.48	[0.44, 0.53]	0.48	[0.44, 0.53]
13. Talk	0.67	[0.63, 0.72]	0.67	[0.63, 0.72]
14. Lonely	0.77	[0.74, 0.81]	0.77	[0.74, 0.71]
15. Unfriendly	0.52	[0.45, 0.58]	0.52	[0.45, 0.58]
17. Cry	0.74	[0.70, 0.78]	0.74	[0.70, 0.78]
18. Sad	0.85	[0.83, 0.88]	0.85	[0.83, 0.88]
19. Dislike	0.68	[0.62, 0.73]	0.68	[0.62, 0.73]
20. Get going	0.70	[0.66, 0.74]	0.70	[0.66, 0.74]
**Correlation of positive** **and negative latent** **factors**	0.41	[0.35, 0.46]	0.41	[0.35, 0.46]
**Direct effects on items** **attributable to French** **language:**				
Item 3. Blues			–0.43	[–0.57, −0.28]
Item 10. Fearful			0.25	[0.12, 0.28]
Item 11. Sleep			–0.30	[–0.44, −0.15]
**Structural effect of** **French language on** **latent factors:**				
French language onpositive factor	–0.08	[–0.25, 0.08]	–0.08	[–0.25, 0.08]
French language onnegative factor	0.09	[–0.07, 0.24]	0.13	[–0.03, 0.28]

a Not corrected for DIF.

b ^b^Corrected for DIF for items 3, 10, and 11.

CI = Confidence Interval.

As shown in Table 2, after correcting for DIF, compared to the base model, there was no change in the difference between English-speaking and French-speaking patients on the “positive” latent factor, and an increase of 0.04 SD in the difference on the “negative” latent factor. The magnitude of this difference was small. Thus, although there was statistically significant DIF on three CES-D items, this did not influence the overall estimates of latent factor scores between English- and French-speaking patients substantively.

As a sensitivity analysis, we ran the MIMIC model with the 17 items that had no statistically significant DIF, yielding virtually the same results as the 20-item model corrected for the 3 DIF items, with a factor loading for language on the positive latent factor of −0.08 (95% CI−0.25 to 0.08) and the negative latent factor of 0.13 (95% CI−0.03 to 0.28).

## Discussion

The main finding of this study was that 3 CES-D items (item 3, *Blues*; item 10, *Fearful*; item 11, *Sleep*) exhibited statistically significant DIF in a sample of English- and French-speaking Canadian SSc patients. However, the magnitude of DIF for the items was small and the effect on overall CES-D scores was negligible. These results suggest that the summed scores of the English and French versions of the measure can be validly compared and pooled among patients with SSc without concern that outcomes will be substantively influenced by differences in scoring metrics between the two versions.

The findings of this study are consistent with those of two previous studies [18], [33]. One study found that the CES-D was essentially measurement equivalent without substantive DIF between English-speaking Canadian and Dutch SSc patients. There was statistically significant, but minor, DIF for items 3, 4 and 7 [18], but DIF did not influence overall estimates substantively. Similarly, in a study of English- and French-speaking Canadian caregivers of people with dementia, statistically significant, but minor, DIF was reported for items 11, 12, 16 and 20 with no substantive influence on overall scores [33].

Differential item functioning, when present, may be related to translational or cultural differences. In the present study, for item 10, no obvious semantic difference between the English and French versions was observed. For item 3, on the other hand, it has been previously noted that items with the English expression “feeling blue” and related expressions such as “having the blues” are difficult to translate because in many languages, including French, a strictly lexical translation for these terms is meaningless [34]–[36]. Thus, in translated versions, the concept needs to be captured with words with sufficient similarity (i.e., *“le sentiment de depression”*), and this might lead to differences between translated versions. For item 11 (*My sleep was restless*), consistent with the present study, a previous study comparing English- and French-speaking Canadian caregivers of persons with dementia found a significant difference between translations [33], but we were not able to identify obvious differences in meaning or intensity of the items between the English and French translations to explain this.

Many studies in Canada routinely integrate data from English and French versions of questionnaires. In addition, international collaborations are increasingly common, and are required to include a sufficient number of patients for adequately powered studies, particularly in rare diseases such as SSc. In SSc, the Scleroderma Clinical Trials Consortium [37] and the EULAR Scleroderma Trials and Research group [38] routinely conduct multicenter drug trials involving patients who complete outcome measures in multiple different languages. In addition, the Scleroderma Patient-centered Intervention Network (SPIN) was recently organized to test psychosocial and rehabilitation interventions in patients from across Canada, the US, and Europe [39], [40]. Thus, future studies should examine the measurement equivalence of frequently used measures central to research in SSc and other medical illnesses.

There are limitations that should be considered in interpreting the results of this study. First, because of the difference in sample size between the English-speaking and French-speaking samples, the core model used to assess DIF relied more on data from English-speaking patients than French-speaking patients. However, since the initial factor analysis yielded the same results in both samples, it does not seem likely that this would have influenced results substantially. Second, our data from both samples were collected from Canadian patients, using a French version of the CES-D that was adapted for use in Quebec. Measurement equivalence could be affected by both language and cultural differences related to the construct being measured. Therefore, it remains to be elucidated to which extend our results generalize to other French-speaking countries. Finally, a potential disadvantage of the MIMIC model, which was used in the present study, compared with other models to assess DIF is that MIMIC does not test for non-uniform DIF, which occurs when the amount of DIF is unequal for different levels of depressive symptoms. An important strength of the MIMIC model, however, is that it allows for adjustment for important covariates that may differ between comparison groups.

An additional limitation relates to the use of the summing of the 4 positive factor items and the 16 negative factor items of the CES-D to arrive at a total score. This is standard practice. However, the low correlation between these two factors (r = 0.41) suggests that summing to get a single general depressive symptom score may be problematic. Other studies have reported similar findings, including studies with cancer patients [41] and community-dwelling older individuals with a high rate of medical illness [42]. In studies of patients with rheumatoid arthritis [43], patients with traumatic brain injury [44], patients undergoing cardiac surgery [45], and HIV+ men [46], on the other hand, somewhat stronger associations between the positive and negative factors have been reported. The reason for the discrepancy in findings is not clear, but whether all 20 items should be summed to a single score merits further investigation. Regardless, the present findings show that the metrics of positive affect scores, negative affect scores, and total scores on the CES-D are essentially equivalent across English and French versions.

In summary, the English and French versions of the CES-D, despite minor DIF, can be used in studies that combine Canadian English- and French-speaking patients with SSc, without undue concern that differences in metrics substantively influence scores.

## Supporting Information

Figure S1 **Items of the French–Canadian CES-D.**
(DOC)
