# Supplementary material for: An Assessment of the Measurement Equivalence of English and French Versions of the Center for Epidemiologic Studies Depression (CES-D) Scale in Systemic Sclerosis
Source: PLoS One. 2014 Jul 18;9(7):e102897. doi: 10.1371/journal.pone.0102897 (PMC4103860; doi:10.1371/journal.pone.0102897)
Supplement: Figure S1 — Items of the French–Canadian CES-D. (DOC) [file pone.0102897.s001.doc]

**Figure S1. Items of the French – Canadian CES-D**

1. J'ai été contrarié(e) par des choses qui habituellement ne me dérangent pas. (*I was bothered by things that usually don't bother me*)
2. Je n'avais pas faim, mon appétit était faible. (*I did not feel like eating; my appetite was poor*)
3. Je ne pouvais pas surmonter le sentiment de dépression, même avec l'aide de ma faimille et de mes amis. (*I felt that I could not shake off the blues even with the help from my family and friends*)
4. Je me sentais aussi bon(ne) que les autres. (*I felt that I was just as good as other people*)
5. J'avais du mal à me concentrer sur ce que je faisais. (*I had trouble keeping my mind on what I was doing*)
6. Je me sentais déprimié(e). (*I felt depressed*)
7. J'avais l'impression que tout ce que je faisais demandait un effort. (*I felt that everything I did was an effort)*
8. J'avais espoir dans l'avenir. (*I felt hopeful about the future*)
9. Je croyais que ma vie était un échec. (*I thought my life had been a failure*)
10. J'étais craintif(ve). (*I felt fearful*)
11. Mon sommeil était agité. (*My sleep was restless*)
12. J'étais heureux(se). (*I was happy*)
13. Je parlais moins que d'habitude. (*I talked less than usual*)
14. Je me sentais seul(e). (*I felt lonely*)
15. Les gens n'étaient pas aimables. (*People were unfriendly*)
16. Je trouvais la vie agreeable. (*I enjoyed life*)
17. J'avais des crises de larmes. (*I had crying spells*)
18. Je me sentais triste. (*I felt sad*)
19. J'avais l'impression que les gens ne m'aimaient pas. (*I felt that people disliked me*)
20. Je n'arrivais pas à "démarrer." (*I could not get "going"*)
